# Identification of continued challenges and opportunities in oncologists' comprehension of immuno-oncology

**DOI:** 10.1186/2051-1426-3-S2-P135

**Published:** 2015-11-04

**Authors:** Tara Herrmann, Frieda Pearce, Charlotte Warren, Haleh Kadkhoda, Lianne Wiggins, Andrea Rindo

**Affiliations:** 1 Medscape Education, New York, NY, USA; 2 Society for Immunotherapy of Cancer, Milwaukee, WI, USA; 3 SITC, Milwaukee, WI, USA

## Background

The treatment of cancer is undergoing a revolution, thanks to new advances in the understanding of the immune system. As part of an ongoing educational curriculum, initial assessment of oncologists' knowledge and practice patterns in the use of immunotherapies identified several challenges. This study's objective was to assess and compare practicing oncologists' comfort level with, and comprehension of, immune-oncologic concepts that play an important role in the care of patients with metastatic cancer.

## Methods

An expert panel was convened to identify knowledge, competence and performance gaps in the area of immuno-oncology (IO). Educational curricula consisting of 6 activities focused on foundational education were developed in 2014 and 2015, and posted online. Intra-activity questions embedded in each program allowed learners to self-report their familiarity with IO concepts, while participants' responses to knowledge- -based questions presented before and after education were collected to measure gains in knowledge. Confidentiality of respondents was maintained. Responses were collected from 5/2014-6/2015.

## Results

Oncologists (N=4,126) who participated in one of the six foundational activities demonstrated on average a 20% improvement from initial baseline in year 1. Year-over-year comparison of oncologists' knowledge of the role of immune checkpoints in the adaptive immune response indicates that a majority of gains observed in year 1, were maintained into the second year of the curriculum. Moreover, additional educational exposure in year 2 of the curriculum allowed recapturing and further augmenting of gains in oncologists' understanding of immune checkpoint regulation.

However, despite the success of the curriculum, oncologists continued to report a lack of familiarity and comfort with IO . Only 11% were very comfortable with prescribing immunotherapies for their cancer patients with advanced disease while 60% characterized themselves as not knowledgeable about CAR T cell therapy. Additional continued clinical challenges that emerged from assessments embedded in specific activities include:

• 25% incorrectly identified CTLA-4, an immune checkpoint, as an activating T cell receptor

• 36-56% failed to understand the role of tumor microenviroment in regulating the adaptive immune response

• 67% did not recognize the mechanism of action and associated long-term activation of CAR T cell therapy

• 59% did not comprehend how to appropriately manage IO treatment-related side effects prior to completing the activity

## Conclusions

This study demonstrates the value of and continued need for appropriately designed online educational interventions on basic concepts of IO. Improvements in competence among oncologists should have beneficial implications for practice and ultimately for patient outcomes.

